# Saponins from *Aralia taibaiensis* Attenuate D-Galactose-Induced Aging in Rats by Activating FOXO3a and Nrf2 Pathways

**DOI:** 10.1155/2014/320513

**Published:** 2014-01-22

**Authors:** Ying-Na Li, Yu Guo, Miao-Miao Xi, Pei Yang, Xue-Ying Zhou, Shuang Yin, Chun-Xu Hai, Jin-Gang Li, Xu-Jun Qin

**Affiliations:** ^1^Department of Geriatrics, The Second Affiliated Hospital of Medicine School, The Xi'an Jiaotong University, Xi'an 710004, China; ^2^Department of Toxicology, The Key Laboratory of Free Radical Biology and Medicine of Shaanxi Province, The Center of Prevention and Treatment on ROS-Induced Injury of FMMU, School of Preventive Medicine, The Fourth Military Medical University, Xi'an 710032, China; ^3^School of Life Science, Shaanxi Normal University, Xi'an 710062, China

## Abstract

Reactive oxygen species (ROS) are closely related to the aging process. In our previous studies, we found that the saponins from *Aralia taibaiensis* have potent antioxidant activity, suggesting the potential protective activity on the aging. However, the protective effect of the saponins and the possible underlying molecular mechanism remain unknown. In the present study, we employed a D-galactose-induced aging rat model to investigate the protective effect of the saponins. We found that D-galactose treatment induced obvious aging-related changes such as the decreased thymus and spleen coefficients, the increased advanced glycation end products (AGEs) level, senescence-associated **β**-galactosidase (SA**β**-gal) activity, and malondialdehyde (MDA) level. Further results showed that Forkhead box O3a (FOXO3a), nuclear factor-erythroid 2-related factor 2 (Nrf2), and their targeted antioxidants such as superoxide dismutase 2 (SOD2), catalase (CAT), glutathione reductase (GR), glutathione (GSH), glutamate-cysteine ligase (GCL), and heme oxygenase 1 (HO-1) were all inhibited in the aging rats induced by D-galactose treatment. Saponins supplementation showed effective protection on these changes. These results demonstrate that saponins from *Aralia taibaiensis* attenuate the D-galactose-induced rat aging. By activating FOXO3a and Nrf2 pathways, saponins increase their downstream multiple antioxidants expression and function, at least in part contributing to the protection on the D-galactose-induced aging in rats.

## 1. Introduction

Aging is a biological process characterized by a progressive deterioration in physiological functions and metabolic processes that leads to morbidity and mortality. Numerous studies have shown that reactive oxygen species (ROS) play a critical role in the aging process since the free radical theory was first proposed by Harman in 1956 [1–4]. Even though there are contradictory reports whether or not increasing antioxidant capacity should increase organismal life span, it is recognized that increasing antioxidant capacity may at least attenuate the degree of aging and the related oxidative damages.

ROS are O_2_-derived active free radicals, such as superoxide anion (O_2_^∙−^), hydroxyl (HO^•^), peroxyl (RO_2_^∙^), and alkoxyl (RO^•^) radicals, as well as nonradical species such as hydrogen peroxide (H_2_O_2_). Physiological levels of ROS are appreciated to function as signaling molecules to regulate a wide variety of physiology (e.g., signal transduction, gene expression, and redox regulation) [5], while high levels of ROS induce oxidative stress. The persistent oxidative stress induces not only the progressive accumulation of oxidative damages, but also the progressive pro-oxidizing shift in the redox state of the cells, resulting in a decline of the functional efficiency of various cellular processes [6]. This is regarded as the key mechanism of ROS-induced aging. On the other hand, mammalian cells have a sophisticated antioxidant system for scavenging ROS to nontoxic forms. This defense system is composed of antioxidant enzymes, such as superoxide dismutase (SOD), catalase (CAT), glutathione reductase (GR), and glutathione peroxidase (Gpx), and nonenzymatic molecules such as glutathione (GSH) and vitamins A, C, and E [7]. It should be noted that certain antioxidant can only scavenge one or several limited kinds of ROS and cannot cope with all kinds of ROS. Elevating the overall antioxidant capacity may be the potentially effective way to reduce the oxidative damage as well as to delay the ROS-accelerated aging process.

In the antioxidant defense system, Forkhead box O3a (FOXO3a) and nuclear factor-erythroid 2-related factor 2 (Nrf2) are two most important transcription factors in regulating multiple antioxidants. FOXO3a is one member of the FOXO family proteins which have been implicated in the regulation of oxidative stress and several other diverse physiologic processes including stress resistance, cell differentiation, cell cycle arrest, metabolism, and apoptosis [8–10]. It has been demonstrated that FOXO3a reduces ROS by the transcriptional activation of SOD2 [9] and CAT [11]. In FOXO family, FOXO3a is the only one which variants have consistently been associated with human longevity in various populations worldwide [12]. Another pivotal role in redox-sensitive proteins transcription is accomplished by Nrf2. Nrf2, which binds to the antioxidant response elements (AREs), has been demonstrated to be a critical transcription factor in the promoter region of a number of genes, encoding for antioxidative and phase 2 enzymes, such as heme oxygenase 1 (HO-1), NAD(P)H-quinone oxidoreductase 1 (NQO1), glutathione S-transferases (GSTs), and glutamate-cysteine ligase (GCL) [13]. Moreover, several lines of evidence suggest that Nrf2 plays a critical role in the regulation of the cellular GSH homeostasis [14]. Recently the decreased Nrf2 expression and activity in aging mammals have been reported by many studies [15–17], and activation of Nrf2 has been proposed as a therapeutic potential target in aging and related diseases [18–20]. Taken together, FOXO3a and Nrf2 offer promising targets enhancing the overall antioxidant capacity to attenuate the aging.

*Aralia taibaiensis* (Araliaceae) is a natural plant widely distributed in the Qinba Mountains of western China. The extract of root bark of *Aralia taibaiensis* has long been used as a folk medicine to treat diabetes, hepatitis, stomach ulcer, and so forth [21]. In our recent studies, we found that the saponins are the main active components of this plant and have potent antioxidant activity *in vitro* [21–23], suggesting the potential protective activity against aging. However, the protective effect of the saponins and the possible molecular mechanism underlying remain unknown.

In the present study, we employed a D-galactose-induced aging rat model to investigate the protective effect of the saponins. We found that D-galactose treatment induced obvious aging-related changes, including the decreased thymus coefficient and spleen coefficient, the increased advanced glycation end products (AGEs) level, the increased senescence-associated *β*-galactosidase (SA*β*-gal) activity, and the increased malondialdehyde (MDA) and H_2_O_2_ levels, on which saponins supplementation showed effective protection. Further results showed that both decreased FOXO3a transcriptional function due to the activation of Akt pathway and the decreased Nrf2 transcriptional function were involved in the D-galactose-induced aging process. By activating Akt/FOXO3a and Nrf2 pathways, saponins supplementation increased the expression and function of their downstream antioxidants, including SOD2, CAT, GR, GSH, GCL, and HO-1, at least in part contributing to the protection on the aging induced by D-galactose.

## 2. Materials and Methods

### 2.1. Preparation and Determination of Total Saponins

The total saponins of *Aralia taibaiensis *were prepared by the method described previously [21] with slight modification. The dry and powdered root bark (20 g) was extracted three times with 80% (v/v) ethanol (herb : ethanol, 1 : 10, w/v) under reflux (80°C) for 60 min. The alcohol extract was concentrated, suspended in distilled water, and then partitioned successively with chloroform (ratio 1 : 3, v/v) and *n-*butanol saturated with water (ratio 1 : 3, v/v, three times). The *n*-butanol extracts were combined and evaporated using a rotary evaporator at 60°C to give a powder residue. The yield was 0.78% (w/w). The content of total saponins was determined approximately using the method described by Hiai et al. [24]. Oleanolic acid was used as a reference standard and the content of total saponins was expressed as oleanolic acid equivalents (OAE), which is 459.30 *μ*g OAE/mg extract.

### 2.2. Animals and Treatment

Male Sprague-Dawley rats, weighing 200–230 g, were obtained from the Laboratory Animal Center of the Fourth Military Medical University. All animal experiments were performed in accordance with the National Institutes of Health Guide for the Care and Use of Laboratory Animals and were approved by the Institutional Animal Ethics Committee of the Fourth Military Medical University. The rats were housed 5 per cage in a climate-controlled room (24-25°C) on a 12 h light/12 h dark schedule with a free access to food and water. All rats were allowed to acclimate for 1 week before starting the experiments. The rats were randomly divided into 4 groups (10 rats in each group): Control group (Control), D-galactose group (D-gal), D-galactose plus saponin group (D-gal+saponin), and saponin group (Saponin). Control group that served as vehicle Control received daily subcutaneous (s.c.) injection of saline (0.9% NaCL) and distilled water orally. D-galactose group and D-galactose plus saponin group received daily s.c. injection of D-galactose at a dose of 150 mg/kg for ten weeks. Meanwhile, D-galactose group served as model received distilled water orally, and D-galactose plus saponin group received crude total saponins of 200 mg/kg in distilled water orally. Saponin group served as the drug Control and received crude total saponins of 200 mg/kg in distilled water orally and s.c. injection of saline.

### 2.3. Thymus and Spleen Coefficients Analysis

After the rats were sacrificed, the weights of thymus and spleen were determined by an accurate electronic balance. The organ coefficient was calculated using the following equation: coefficient (g/100 g) = organ weight/body weight × 100.

### 2.4. AGEs Level Analysis

The AGEs level in serum was determined by an ELISA kit (CUSABio Biotech, Wuhan, China). Briefly, serum was diluted 2 times in standard diluent buffer. Aliquots of 100 *μ*L of diluted serum were added to the appropriate microtiter wells provided by the kit. For the standard curve, 100 *μ*L of different concentration AGEs standards and the blank standard were added to the appropriate microtiter wells. The plate was incubated for 2 hours at 37°C. After discarding the liquid of each well, 100 *μ*L of biotinylated antibody working solution was added into each well and incubated for 1 hour at 37°C. After washing, 100 *μ*L streptavidin-HRP working solution was added to each well and incubated for 1 hour at 37°C. Lastly, 90 *μ*L of stabilized chromogen was added to each well and incubated for 30 minutes at 37°C in the dark before the addition of 50 *μ*L of stop solution. The absorbance at 450 nm was recorded on a plate reader. A standard curve was generated and the concentrations for unknown samples were obtained from the standard curve.

### 2.5. SA*β*-Gal Staining Analysis

After the animal was anesthetized and dissected, the liver was removed and immediately frozen in liquid nitrogen, and 10-*μ*m frozen sections were cut by a Leica CM1900 freezing microtome (Leica, German). The frozen sections were washed with PBS and then incubated overnight in SA*β*-gal staining solution (Beyotime Institute of Biotechnology, Haimen, China) at 37°C. The following day, the samples were washed with PBS and coverslipped for immediate imaging with a BX51 Olympus microscope (Olympus, Japan).

### 2.6. Tissue Homogenate Preparation

After the rats were sacrificed, the livers were quickly removed and placed in ice-cold buffer (50 mM potassium phosphate buffer, pH 7.4). Then, 10% (w/v) tissue homogenates were prepared with a Teflon homogenizer. The crude homogenates were centrifuged at 5000 g for 10 min. The supernatants were collected for biochemical assays. All procedures were performed at 4°C.

### 2.7. Malondialdehyde (MDA) and Hydrogen Peroxide (H_**2**_O_**2**_) Analysis

The levels of MDA in serum and liver tissue were determined as previously described [25]. The H_2_O_2_ level in liver was determined by the Amplex Red Hydrogen Peroxide Assay Kit from Invitrogen, strictly following the protocol instructions provided with the kit.

### 2.8. Antioxidants Analysis

The activities of SOD, CAT, and GR were determined by the kits from Beyotime Institute of Biotechnology (Haimen, China), respectively. The GSH levels were determined by the kit from Nanjing Jiancheng Institute of Biotechnology (Nanjing, China). All experimental procedures were carried out in strict accordance with protocol instructions provided by the manufacturers.

### 2.9. Protein Extracts Preparation

One hundred milligrams of liver tissue was homogenized by a glass homogenizer in 1 mL cold RIPA buffer containing 1% protease inhibitor cocktail and 1% phosphatase inhibitor cocktail (Sigma-Aldrich, St. Louis, MO). After standing on ice for 30 minutes, the homogenate was centrifuged at 14,000 g for 20 minutes at 4°C. The supernatant was collected as the total protein extract for western blot assay. Nuclear extract was prepared using the NE-PER nuclear and cytoplasmic extraction kit from Pierce Biotechnology (Pierce, Rockford, ILL) following the protocol instructions provided with the kit. Protein concentrations were determined by the Pierce BCA Protein Assay Kit (Pierce).

### 2.10. Electrophoretic Mobility Shift Assay (EMSA) of FOXO3a Activity

The FOXO3a transcriptional activity was determined by EMSA method as described by Choi et al. [26]. Oligonucleotide for FOXO3a binding was 5′-TTA GTC ATTTTG TTT GTT CAT A-3′. The binding activities of FOXO3a in nuclear extracts (10 *μ*g) was detected by the LightShift Chemiluminescent EMSA kit (Pierce) and the Chemiluminescent Nucleic Acid Detection Module (Pierce, Rockford, ILL) according to the instructions of the manufacturers. The signal were detected using the SuperSignal West Pico Chemiluminescence kit (Pierce) on a Bio-Rad ChemiDocXRS Gel Imaging System.

### 2.11. Western Blot Analysis

Total protein extract (30 *μ*g of protein) or nuclear extract (10 *μ*g of protein) from liver was resolved on an 8–10% sodium dodecyl sulfate (SDS) polyacrylamide gel and transferred onto nitrocellulose membrane (Bio-Rad, Hercules, CA) and incubated for 2 hours in tris-buffered saline with Tween (TBST) (10 mM Tris, pH 8.0, 150 mM NaCL, and 0.1% Tween 20) containing 5% nonfat milk at room temperature. The membrane was then incubated overnight at 4°C with the following primary antibodies, SOD2 (1 : 5000, Epitomics), SOD1 (1 : 10000, Epitomics), CAT (1 : 1000, Epitomics), FOXO3a (1 : 1000, Epitomics), p-FOXO3a (ser253) (1 : 1000, Epitomics), HO-1 (1 : 2000, Epitomics), GCLC (1 : 5000, Bioworld), GCLM (1 : 1000, Epitomics), Nrf2 (1 : 500, Santa Cruz Biotechnology), p-Nrf2 (1 : 1000, Bioss), Akt and p-Akt (1 : 1000, Cell Signaling), Lamin B (1 : 500, Epitomics), and *β*-actin (1 : 500, Santa Cruz Biotechnology). After washing with TBST, the membrane was then incubated for 1 hour with horseradish peroxidase conjugated secondary antibody (all from Invitrogen with a dilution of 1 : 5000), and signal was detected using the SuperSignal West Pico Chemiluminescence kit on a Bio-Rad ChemiDocXRS Gel Imaging System. The band intensities were quantified by Quantity One Software version 4.6.3. Protein levels were normalized to *β*-actin or Lamin B as compared with the Control (set to 1).

### 2.12. Data Analysis

Data are presented as means ± SD. Intergroup comparisons were performed by one-way analysis of variance (ANOVA) (if data followed normal distribution), or by nonparametric methods, namely, Kruskal-Wallis rank-sign analysis of variance and the Mann-Whitney test (if data did not follow normal distribution). A value of *P* < 0.05 was considered statistically significant.

## 3. Results

### 3.1. Saponins from *Aralia taibaiensis* Attenuate D-Galactose-Induced Aging in Rats

**Figure 1:**
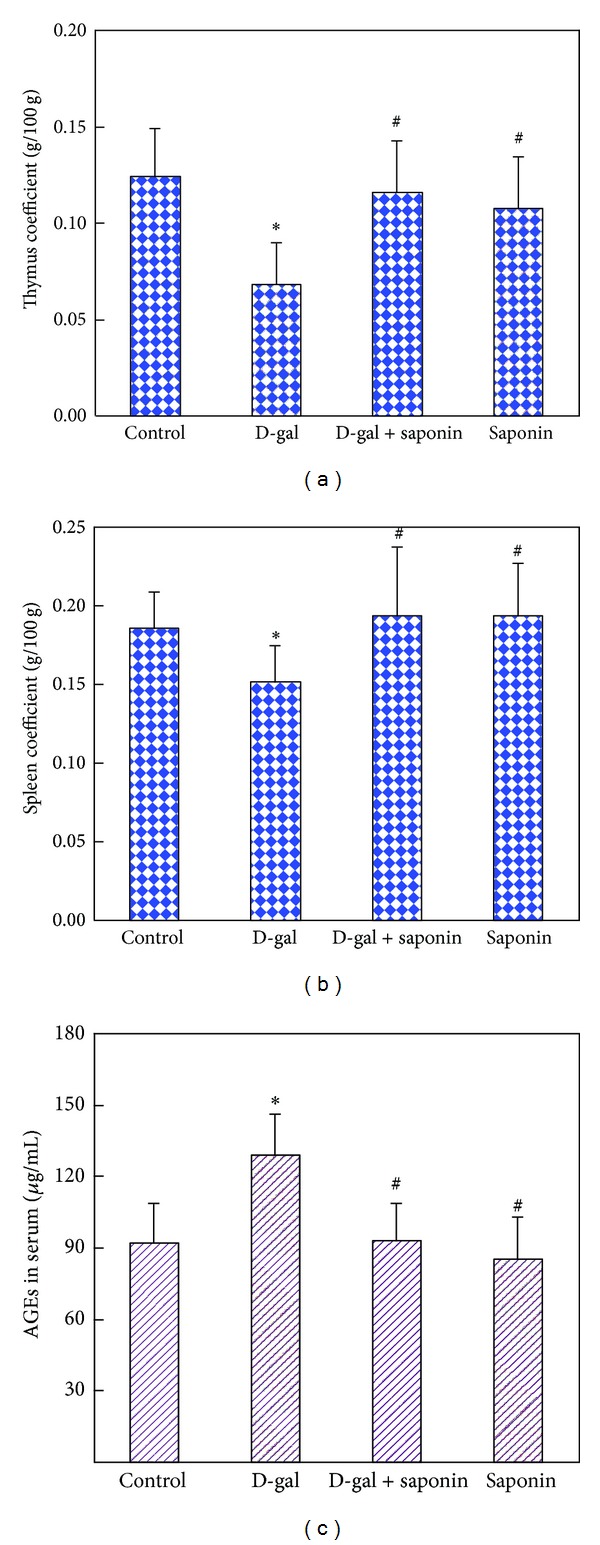
Saponins supplementation attenuated the decreased thymus and spleen coefficients as well as the increased AGEs level in D-galactose-induced aging rats. (a) Thymus coefficient, (b) spleen coefficient, and (c) AGEs level in serum. Data are expressed as mean ± SD; *n* = 10. Intergroup comparisons were performed by Kruskal-Wallis rank-sign analysis of variance and the Mann-Whitney test. *P < 0.05 versus Control and ^#^ *P* < 0.05 versus D-gal.

**Figure 2:**
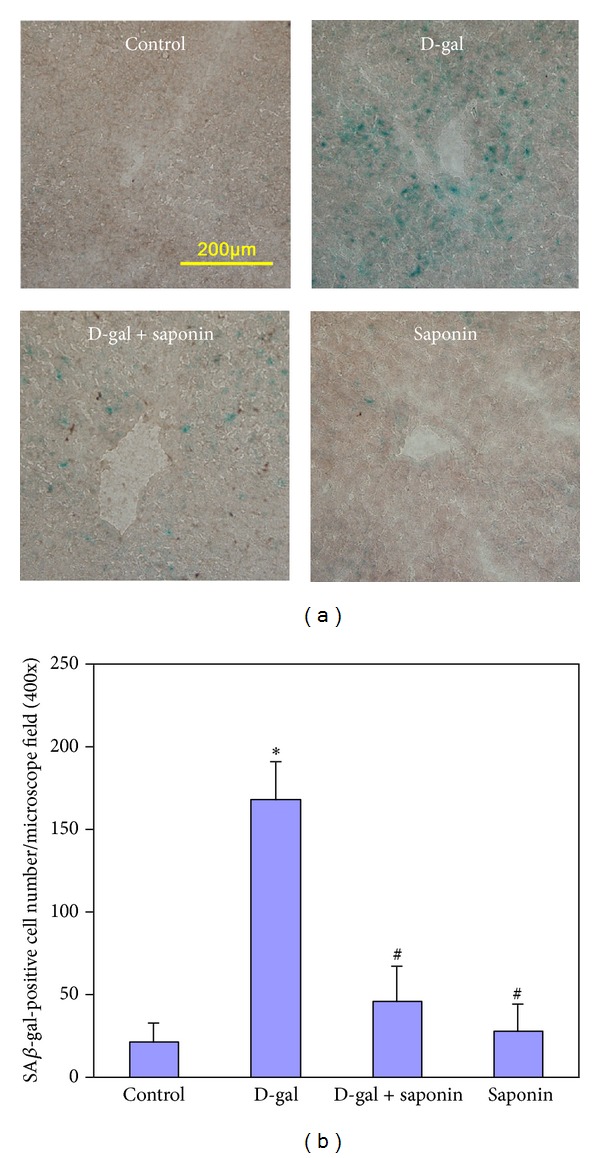
Saponins supplementation attenuated the increased activity of SA*β*-Gal in liver of D-galactose-induced aging rats. (a) Representative image of SA*β*-gal staining in each group was captured with a BX51 Olympus microscope. Blue staining means SA*β*-gal-positive cells. (b) Quantification of the SA*β*-gal-positive cell number. Data are expressed as mean ± SD; *n* = 10. Intergroup comparisons were performed by ANOVA. *P < 0.05 versus Control and ^#^ *P* < 0.05 versus D-gal.

D-galactose-induced aging rat model has been demonstrated to be reliable to mimic the natural aging [27, 28]. It is widely used for studying aging mechanisms and screening drugs to attenuate the aging [29]. In mammals, atrophy of immune organs with age results in the decreases of the immune organ coefficients, such as thymus coefficient and spleen coefficient, which are often used as hallmarks at organ level to evaluate the success of the aging animal model. In the present study, ten weeks of D-galactose treatment induced obvious decreases of thymus coefficient and spleen coefficient, suggesting the success of the aging rat model. Saponins supplementation significantly antagonized these changes induced by D-galactose treatment, suggesting the effective protection of saponins (Figures 1(a) and 1(b)). AGEs are the end results of a chain of chemical reactions involving an initial glycation reaction. Besides, in diabetes, AGEs are formed as a result of normal metabolism and aging. High level of AGEs in serum is frequently recognized as an aging related biomarker. In the present study, D-galactose treatment significantly increased the AGEs level in serum, on which saponins supplementation showed effective inhibition (Figure 1(c)), also suggesting the protection of saponins on the D-galactose-induced aging. In order to confirm the success of the aging model and the protection of saponins, we chose the liver as the representative organ to detect the most widely recognized aging biomarker, SA*β*-gal activity [30]. D-galactose treatment greatly increased the SA*β*-gal activity (represented by the blue staining in the images, Figure 2), confirming the aging change of the liver, on which saponins supplementation showed effective inhibition, also confirming the protection of saponins. These results show that, in the present study, the D-galactose-induced aging rat model is successful and saponins from *Aralia taibaiensis* effectively attenuate D-galactose-induced aging in rats.

### 3.2. Saponins Inhibited D-Galactose-Induced Oxidative Stress by Elevating Multiple Antioxidants in Rats

**Figure 3:**
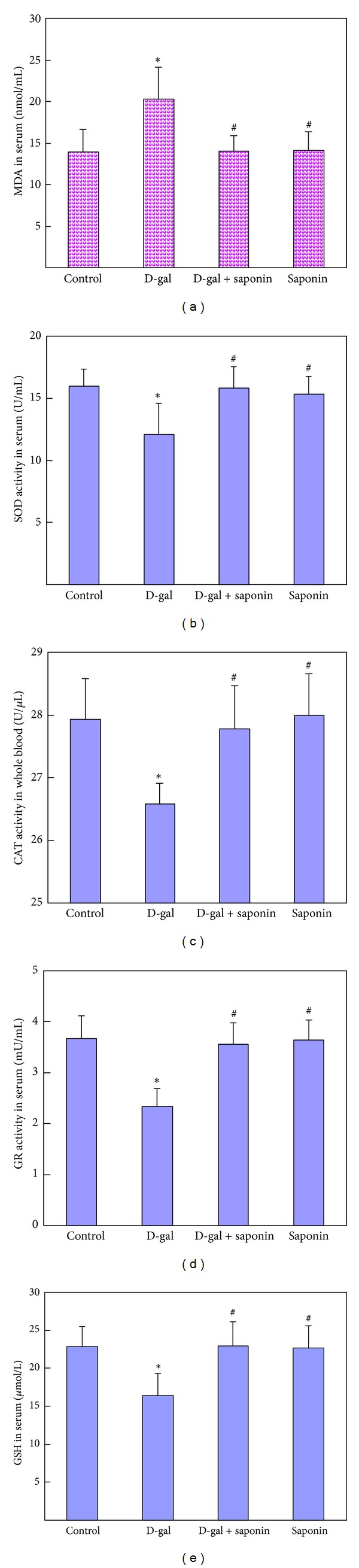
Saponins supplementation attenuated the increased level of MDA (a) and the decreased activities of SOD (b), CAT (c), GR (d), and as well as the level of GSH (e) in serum of D-galactose-induced aging rats. Data are expressed as mean ± SD; *n* = 10. Intergroup comparisons were performed by ANOVA. *P < 0.05 versus Control and ^#^ *P* < 0.05 versus D-gal.

**Figure 4:**
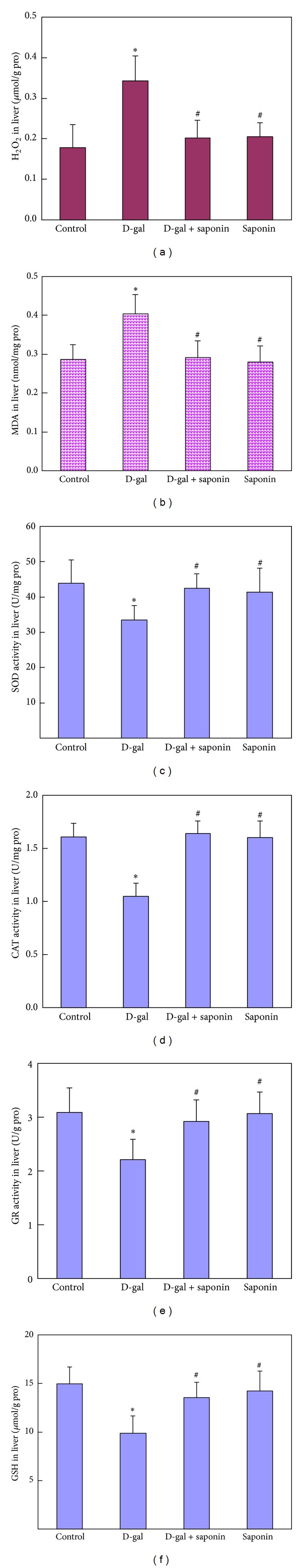
Saponins supplementation attenuated the increased level of H_2_O_2_ (a) and MDA (b), and the decreased activities of SOD (c), CAT (d), GR (e), and the level of GSH (f) in liver of D-galactose-induced aging rats. Data are expressed as mean ± SD; *n* = 10. Intergroup comparisons were performed by ANOVA. *P < 0.05 versus Control and ^#^ *P* < 0.05 versus D-gal.

As ROS play an important role in the aging process and stimulating ROS production is reported as one of the most important mechanisms underlying the D-galactose induced aging [27, 31, 32], we firstly detected most recognized oxidative damage product, MDA. D-galactose treatment greatly increased the MDA level in serum, confirming D-galactose-induced oxidative stress reported by others [27, 33, 34]. Saponins supplementation significantly inhibited the D-galactose increased MDA (Figure 3(a)), suggesting that saponins attenuated the aging by inhibiting oxidative stress. Next we detected several key antioxidants, including SOD, CAT, GR, and GSH. D-galactose treatment decreased the activities of SOD, CAT, and GR as well as the content of GSH in the serum, on which saponins supplementation showed effective protection (Figures 3(b), 3(c), 3(d), and 3(e)), suggesting that saponins may inhibit D-galactose-induced oxidative stress by elevating these antioxidants. Again we chose liver as the representative organ to detect the oxidative stress related parameters. D-galactose treatment greatly increased the levels of H_2_O_2_ and MDA and decreased the activities of SOD, CAT, and GR as well as the content of GSH in livers, while saponins supplementation significantly inhibited these changes (Figure 4), confirming the antioxidative protection of saponins. These results together with those in serum suggest that oxidative stress plays a critical role in the D-galactose-induced aging, and saponins supplementation may exert its protective effect by elevating multiple antioxidants and inhibiting D-galactose-induced oxidative stress.

### 3.3. FOXO3a Pathway Is Involved in the Protection of Saponins on the D-Galactose-Induced Rat Aging

**Figure 5:**
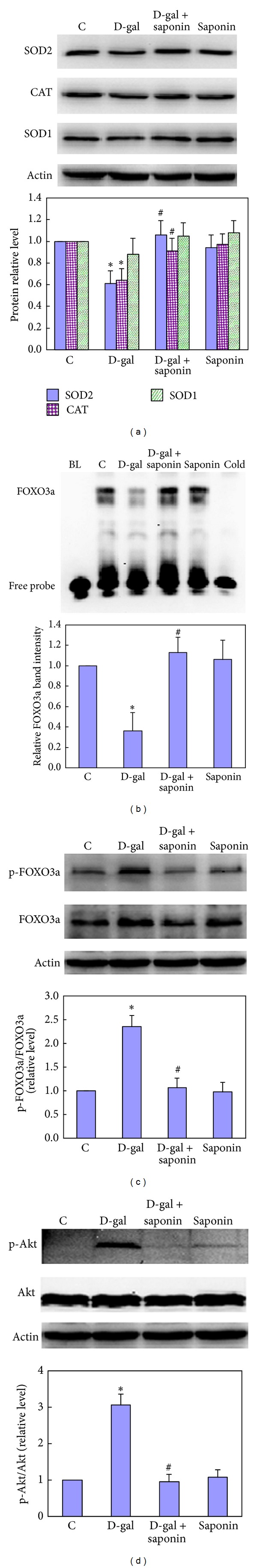
Saponins supplementation activated FOXO3a pathway in liver of D-galactose-induced aging rats. The protein levels of SOD2, CAT, and SOD1 were determined by Western blot (a). The transcriptional activity of FOXO3a was determined by EMSA (b). The protein levels of p-FOXO3a (S253) (c) and p-Akt (d) were also determined by Western blot. The band intensities were quantified by Quantity One Software version 4.6.3. Protein levels were normalized to *β*-actin as compared with the untreated Control (set to 1). Data are expressed as mean ± SD; *n* ≥ 3. Intergroup comparisons were performed by ANOVA. *P < 0.05 versus Control and ^#^ *P* < 0.05 versus D-gal.

Biochemical assay results (Figures 3 and 4) showed that both SOD and CAT functions were involved in the protective effect of saponins. In order to make clear if SOD and CAT were regulated at transcriptional level, we detected the protein levels of these antioxidant enzymes by Western blot. As shown in Figure 5(a), D-galactose treatment decreased the protein levels of SOD2, CAT, and SOD1, on which saponins supplementation showed significant protection, suggesting that transcriptional regulation of SOD2 and CAT was involved in the protection process of saponins. As FOXO3a is a transcription factor in the upstream directly regulating SOD2 and CAT expressions [9, 11], we further detected the FOXO3a transcriptional activity by EMSA. Consistent to the results of SOD2 and CAT, D-galactose treatment remarkably decreased the FOXO3a activity, on which saponins supplementation showed significant protection (Figure 5(b)), suggesting that FOXO3a activity was inhibited by D-galactose treatment and retrieved by the saponins supplementation during this process. Normally, FOXO3a binds on the DNA and transcriptionally induces the targeted gene expressions. Upon FOXO3a phosphorylation by Akt in the upstream, it will be exported to the cytoplasm and lose its transcriptional function [35, 36]. So we next detected the phosphorylation of FOXO3a (p-FOXO3a S253) and the activation of Akt pathway. As shown in Figures 5(c) and 5(d), D-galactose treatment significantly increased the levels of both p-FOXO3a (S253) and p-Akt, on which saponins supplementation showed effective inhibition. These results indicate that Akt/FOXO3a pathway is involved in the D-galactose-induced aging process, and saponins may show protective effect by activating Akt/FOXO3a pathway and enhancing expression of its downstream antioxidants, such as SOD2 and CAT.

### 3.4. Nrf2 Pathway Is Another Target of Saponins in the Protection of D-Galactose-Induced Rat Aging

**Figure 6:**
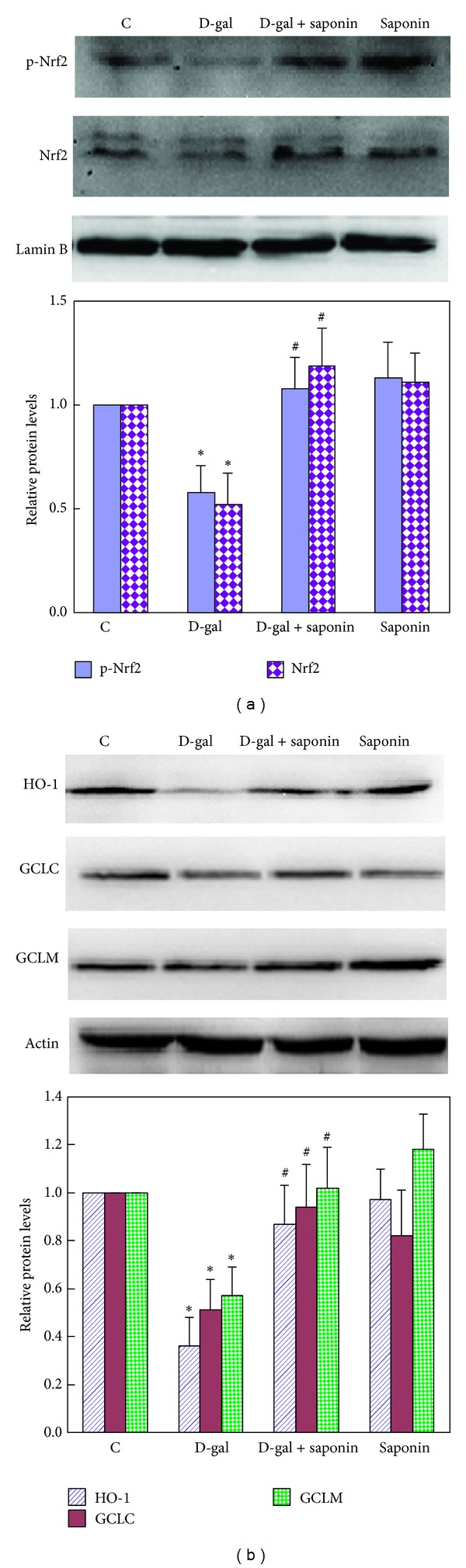
Saponins supplementation activated Nrf2 pathway in liver of D-galactose-induced aging rats. The protein levels of p-Nrf2 and Nrf2 (a), as well as its targeted genes HO-1, GCLC, and GCLM (b), were determined by Western blot. The band intensities were quantified by Quantity One Software version 4.6.3. Protein levels were normalized to *β*-actin or Lamin B as compared with the untreated Control (set to 1). Data are expressed as mean ± SD; *n* ≥ 3. Intergroup comparisons were performed by ANOVA. *P < 0.05 versus Control and ^#^ *P* < 0.05 versus D-gal.

The results in Figures 3 and 4 showed that the GSH was another important antioxidant regulated by saponins in the protective process. As GSH has been demonstrated to be regulated by another pivotal transcription factor, Nrf2 [14], we speculated that Nrf2 was also involved in the protective process of saponins. In order to test this hypothesis, we detected the protein accumulations of Nf2 as well as its active phosphorylated form p-Nrf2 in the nucleus. As shown in Figure 6(a), D-galactose treatment significantly inhibited the protein accumulations of both Nrf2 and p-Nrf2, on which saponins supplementation showed effective protection. This result indicates the involvement of Nrf2 pathway in the protective process of saponins. In order to confirm this, we further detected the expressions of Nrf2 targeted antioxidants in the downstream. As shown in Figure 6(b), D-galactose treatment significantly decreased the protein levels of HO-1, catalytic subunit of GCL (GCLC), and modifier subunit of GCL (GCLM), on which saponins supplementation showed effective protection. These changes of antioxidant were consistent with the changes of Nrf2 and p-Nrf2 shown in Figure 6(a), demonstrating the activation of Nrf2 pathway by saponins. These results suggest Nrf2 pathway may be another target of saponins in the protective process. By enhancing the Nrf2 transcription function, saponins supplementation increases the multiple antioxidants in the downstream, such as HO-1, GCLM, GCLC, GR, and GSH, contributing to the attenuated oxidative stress and aging.

## 4. Discussion

Aging is a natural process of life. Even though the aging is inevitable, appropriate strategies may delay the aging process. One of the most promising ways is to improve the antioxidant capacity due to the critical role of ROS in the aging. In our previous *in vitro* studies, we found that saponins from *Aralia taibaiensis *showed potent antioxidant activity [21–23]. In the present study, for the first time, we demonstrated that saponins from* Aralia taibaiensis *attenuated D-galactose induced aging in rats. Further results showed that both FOXO3a pathway and Nrf2 pathway were involved in the protective process. By activating FOXO3a and Nrf2 pathways, saponins supplementation enhanced multiple key antioxidants and decreased oxidative stress, at least in part contributing to the protection on D-galactose induced aging in rats.

It is well known that antioxidant supplementation may reduce oxidative stress and potentially benefit oxidative related diseases. However, it should be noted that single antioxidant may clear only one or several limited kinds of ROS. For instance, SOD converts O_2_^∙−^ to H_2_O_2_, which is still toxic, and needs further to be converted to H_2_O with the help of CAT or Gpx. So enhancing multiple antioxidants at the same time may be the most potent strategy to truly reduce oxidative stress and potentially delay the aging process. In the present study, saponins supplementation significantly increased multiple antioxidants inhibited by D-galactose treatment, including SOD2, CAT, GR, GSH, HO-1, GCLC, and GCLM. The overall elevation of the antioxidant network may result in the reduced ROS and oxidative damages (Figures 3(a), 4(a), and 4(b)), contributing to the effective protection against D-galactose-induced aging (Figures 1 and 2).

The remaining question is how the saponins increase multiple antioxidants at the same time. One possibility is that saponins increase these antioxidants by targeting activation of their common transcription factors in the upstream. FOXO3a is one of the key transcription factors in the antioxidant defense system [37]. As the most important member related to aging in FOXO family, FOXO3a has emerged as a convergence point of signaling in response to growth factor stimulation and oxidative stress and plays a critical role in a wide variety of cellular outputs, including glucose metabolism, cell cycle arrest, differentiation, apoptosis, and ROS detoxification [38, 39]. Normally, FOXO3a binds on DNA and directly promotes series of gene expressions, such as SOD2, CAT, p27kip1, and GADD45 [9, 11]. In the present study, the synchronous decreases or increases of SOD2 and CAT protein levels by D-galactose treatment or saponins supplementation (Figure 5(a)) indicated the involvement of FOXO3a. Indeed, transcriptional activity assay by EMSA demonstrated that the FOXO3a activity was inhibited by D-galactose treatment, on which saponins supplementation showed effective protection (Figure 5(b)), demonstrating that FOXO3a was one target of saponins in the protective process. Literature has shown that FOXO3a is negatively regulated by Akt-dependent phosphorylation at three specific sites (Thr32, Ser253, and Ser315), and phosphorylated FOXO3a will bind to 14-3-3 protein and be exported from the nucleus to the cytoplasm, thereby losing its transcriptional function [36, 39]. So we speculated that Akt-dependent phosphorylation of FOXO3a may be involved in the depressed FOXO3a function. This hypothesis was confirmed by the increased p-FOXO3a (S253) level as well as the increased p-Akt level (Figures 5(c) and 5(d)). Taken together, these results indicate that Akt-dependent phosphorylation of FOXO3a is involved in the D-galactose-induced aging process, and Akt/FOXO3a pathway is the possible target of saponins in promoting the multiple antioxidants to protect from the D-galactose-induced aging.

GSH is an important intracellular antioxidant and redox potential regulator that plays a vital role in drug detoxification and elimination and in cellular protection from damage by free radicals, peroxides, and toxins [40]. During the process of ROS detoxification, GSH is converted to oxidized glutathione (GSSG) and converted back to GSH by GR [41]. The biosynthesis of GSH is rate limiting catalyzed by GCL composed of GCLC and GCLM [42, 43]. GR and GCL collaborate to maintain the sufficient GSH level in the body. In the present study, D-galactose treatment decreased GSH level and GR activity as well as GCLC and GCLM protein levels, on which saponins supplementation showed effective protection (Figures 3, 4, and 6), suggesting the involvement of GSH and related enzymes in the protective effect of saponins. GCLC and GCLM are classic targeted genes directly regulated by Nrf2, and recent study demonstrates that GR is also induced after Nrf2 binding to AREs under oxidative stress [14]. The synchronous changes of GCLC, GCLM, and GR strongly suggested the involvement of Nrf2 in the protective process of saponins, which was confirmed by the changes of Nrf2 and p-Nrf2 protein level in nucleus (Figure 6). Besides GCLC and GCLM, HO-1 is another important antioxidant protein directly regulated by Nrf2. HO-1, a ubiquitous inducible cellular stress protein, serves a major metabolic function as the rate-limiting step in the oxidative catabolism of heme, leading to formation of equimolar amounts of biliverdin (BV), Fe^2+^, and carbon monoxide (CO) [44, 45]. BV formed in this reaction is rapidly converted to the strong antioxidant bilirubin (BR) by the action of biliverdin reductase. The potent antioxidant effects of BV and BR are thought to contribute in a significant manner to the overall protective effect of HO-1 [46, 47]. In the present study, the consistent change of HO-1 protein level with Nrf2 not only confirms the involvement of Nrf2 pathway, but also suggests the critical role of HO-1 in the antioxidative protection of the saponins. These results indicate Nrf2 as another target of the saponins in the protective process against D-galactose-induced aging. It should be noted that, normally, Nrf2 binds to Kelch-like ECH-associated protein 1 (Keap1) in the cytoplasm, and oxidative stress may modify the cysteines of Keap1 leading to nuclear translocation of Nrf2 and subsequent induction of target gene expressions [48, 49]. The induction of Nrf2 translocation by ROS is reported by different studies [50–52]. However, in the present study, D-galactose-induced oxidative stress decreased Nrf2 translocation and its targeted antioxidants expression. One possible explanation is the different response of Nrf2 to acute or chronic oxidative stress. Ten weeks of D-galactose treatment in the present study induced more like chronic than acute oxidative stress. The decline of Nrf2 protein level and its transcription function is consistent with the change of Nrf2 in the natural aging animals [16, 17, 53]. How chronic D-galactose treatment inhibiting Nrf2 function needs further study in the future. Taken together, these results suggest that Nrf2 function is inhibited during the D-galactose-induced aging process. By activating Nrf2, saponins supplementation increases Nrf2 targeted multiple antioxidants, at least in part contributing to the protection against the D-galactose-induced aging.

In conclusion, by employing the D-galactose-induced aging rat model, we demonstrate that saponins from *Aralia taibaiensis* attenuate D-galactose-induced aging in rats. Both FOXO3a and Nrf2 pathways are involved in the process. By activating FOXO3a and Nrf2, saponins increase the expression and function of their targeted multiple antioxidants, at least in part contributing to the protection on the D-galactose-induced aging. This study, for the first time, demonstrates the activity of saponins from *Aralia taibaiensis* on the D-galactose-induced aging and also indicates the potential underlying molecular mechanism. Moreover, it give us a clue that enhancing overall antioxidant capacity by targeting the key antioxidant transcription factors at the same time is a promising strategy in exploring the method and medicine to attenuate the aging process. It should be noted that, in this study, it is still unknown whether the saponins protect from the D-galactose-induced aging by specific targeting FOXO3a and Nrf2 or by indirect regulating FOXO3a and Nrf2 due to their antioxidant activity. Further studies should be done to answer this important question in the future.
